# Neuroimmune communication in allergic rhinitis

**DOI:** 10.3389/fneur.2023.1282130

**Published:** 2023-12-21

**Authors:** Yi Zhou, Ru Chen, Lili Kong, Yaoyao Sun, Jing Deng

**Affiliations:** ^1^Department of Otolaryngology, Jiaxing University Master Degree Cultivation Base, Zhejiang Chinese Medical University, Zhejiang, China; ^2^Department of Otolaryngology, The First Hospital of Jiaxing, Jiaxing, China

**Keywords:** allergic rhinitis (AR), neuroimmune communication, neuropeptides, neurogenic inflammation, nasal hyperresponsiveness (NHR), neuroimmune cell units

## Abstract

The prevalence rate of allergic rhinitis (AR) is high worldwide. The inhalation of allergens induces AR, which is an immunoglobulin E-mediated and type 2 inflammation-driven disease. Recently, the role of neuroimmune communication in AR pathogenesis has piqued the interest of the scientific community. Various neuropeptides, such as substance P (SP), vasoactive intestinal peptide (VIP), calcitonin gene-related peptide (CGRP), nerve growth factor (NGF), and neuromedin U (NMU), released via “axon reflexes” or “central sensitization” exert regulatory effects on immune cells to elicit “neurogenic inflammation,” which contributes to nasal hyperresponsiveness (NHR) in AR. Additionally, neuropeptides can be produced in immune cells. The frequent colocalization of immune and neuronal cells at certain anatomical regions promotes the establishment of neuroimmune cell units, such as nerve-mast cells, nerve-type 2 innate lymphoid cells (ILC2s), nerve-eosinophils and nerve-basophils units. Receptors expressed both on immune cells and neurons, such as TRPV1, TRPA1, and Mas-related G protein-coupled receptor X2 (MRGPRX2) mediate AR pathogenesis. This review focused on elucidating the mechanisms underlying neuroimmune communication in AR.

## 1 Introduction

Allergic rhinitis (AR), a highly prevalent atopic disorder, is associated with immunoglobulin E (IgE) antibody-mediated immune responses (1). Globally, AR affects up to 40% of the human population. Recent studies have focused on the diagnosis, pathophysiological mechanisms, epidemiology, treatment, and comorbidities of AR. Previous studies have reported the correlation between AR and asthma. AR and asthma, which are characterized by similar inflammatory reactions, are associated with enhanced responses to various inhalants (2). Moreover, AR is an independent risk factor for the development of asthma. In particular, 40% of patients with AR have or will develop asthma. As the inflammatory responses in the upper and lower airways are similar and interconnected in AR and asthma, they can be considered “one airway, one disease” (3). The mutual effects of AR and asthma have been previously reported. Additionally, allergic conjunctivitis may also be associated with AR. Nasal symptoms, which are characterized by mucosal inflammation and hyperresponsiveness, may be induced by the hyperresponsiveness of the upper airways after allergen inhalation (2, 4–6).

The submucosa or subepithelial region of the nose comprises abundant immune cells that contribute to allergic inflammation, which is also regulated by neuroimmune-derived factors (7). The exposure to an allergen promotes the cross-linking of mucosal mast cell (MC) and basophil surface-bound IgE with its receptor and rapidly induces nasal symptoms in sensitized individuals through degranulation and the release of various chemical mediators, such as histamine, prostaglandin D2, and cysteinyl leukotrienes (5, 8, 9). Eosinophils in the nasal mucosa are also activated during this process. Nasal epithelial injury exposes nerve fibers and promotes the release of granule contents, which elicit T helper 2 cell (Th2) response, resulting in the recruitment of eosinophils, basophils, and T cells to sustain the inflammatory response (8). Th17 and regulatory T (Treg) cells, rather than the traditional Th1/Th2 balance paradigm, were recently reported to mediate AR pathogenesis (10). Substance P (SP) and calcitonin gene-related peptide (CGRP) regulate Th17/Treg differentiation (11, 12). These reactions result in vasodilation, increased vascular permeability, and mucus production, as well as the stimulation of sensory nerves, inducing typical symptoms, such as itching, sneezing, watery rhinorrhea, and nasal congestion and impairing the quality of life (QOL) (13). As neuronal activity is involved in the induction of these symptoms, AR can be characterized as a neuroimmune disorder.

Neuroimmunology, a biological science field, is an area of active research. Bidirectional interactions between the nervous system and the immune system involve the following four basic components: neuromediators secreted by the neurons; immune factors released by the immune cells; cytokines released by the neuroendocrine cells and neuropeptides released by the immune cells; common receptors expressed in the immune cells and neurons (14, 15). Recent studies have reported the role of neuroimmune interactions in the development of allergic diseases, such as atopic dermatitis, prurigo nodularis, AR, asthma, food allergy, chronic rhinosinusitis with nasal polyps, eosinophilic esophagitis, allergic rhinoconjunctivitis, and urticaria (16–20). Thus, the processes involved in neuroimmune communication must be elucidated to improve our understanding of AR pathogenesis.

This review summarizes neuroimmune communication in AR and highlights the role of the immune system-nervous system interaction in AR pathogenesis. The review of known neuroimmunomodulation mechanisms of AR provides a potential research direction for exploring therapeutic strategies to target neuroimmune interaction. Additionally, this review can serve as a theoretical foundation for the application of these therapeutic strategies and provide useful insights for the development of novel diagnostic and therapeutic strategies for AR.

## 2 Neurogenic inflammation and AR nasal hyperresponsiveness

### 2.1 The sensory neuron of nasal mucosa and reflex hyperresponsiveness

Nociceptors are specialized sensory neurons that detect and respond to noxious or potentially noxious stimuli, such as mechanical, chemical, or thermal stimuli. Additionally, nociceptors of afferent nerves are highly susceptible to stimulation during an acute allergic reaction (21). These nerve terminals in the human nasal cavity are the peripheral processes of the trigeminal ganglion-based primary sensory neurons (22). Generally, human nasal nociceptors are C-fibers, which are typically sensitive to chemical and physical stimulation (multimode) (22). These sensory endings express several receptors and ion channels, including transient receptor potential (TRP) channels (such as TRPV1 and TRPA1), G protein-coupled receptors (GPCRs), acid-sensing ion channels, mechano-sensitive channels, voltage-gated ion channels, and purinergic receptors, which convert environmental signals into electrical signals (22, 23).

NHR, which is observed in 60–70% of patients with AR, is associated with alterations in the following two types of innervating nasal nerves: afferent nerves (somatosensory system) and efferent nerves (sympathetic or parasympathetic motor system). Some nasal symptoms, such as sneezing, rhinorrhea, itching, or obstruction may develop in a hyperresponsive nasal cavity in response to various stimuli, such as cold dry air, capsaicin, hyperosmolar saline, and bradykinin with markedly enhanced nasal reflexes (1, 6, 22, 24). The application of afferent C-fiber activators to the nasal mucosa in animal models and in humans increases reflex hypersensitivity, markedly enhancing NHR (25). In addition to the nasal mucosa, reflex hypersensitivity also affects other areas of the airways in humans (26). However, not all NHR symptoms in patients are directly caused by neuronal malfunction, some of them may even be brought on by immune cells (22).

### 2.2 “Axon reflexes” and “central sensitization” associated with “neurogenic inflammation” of nasal hyperresponsiveness

**Figure 1:**
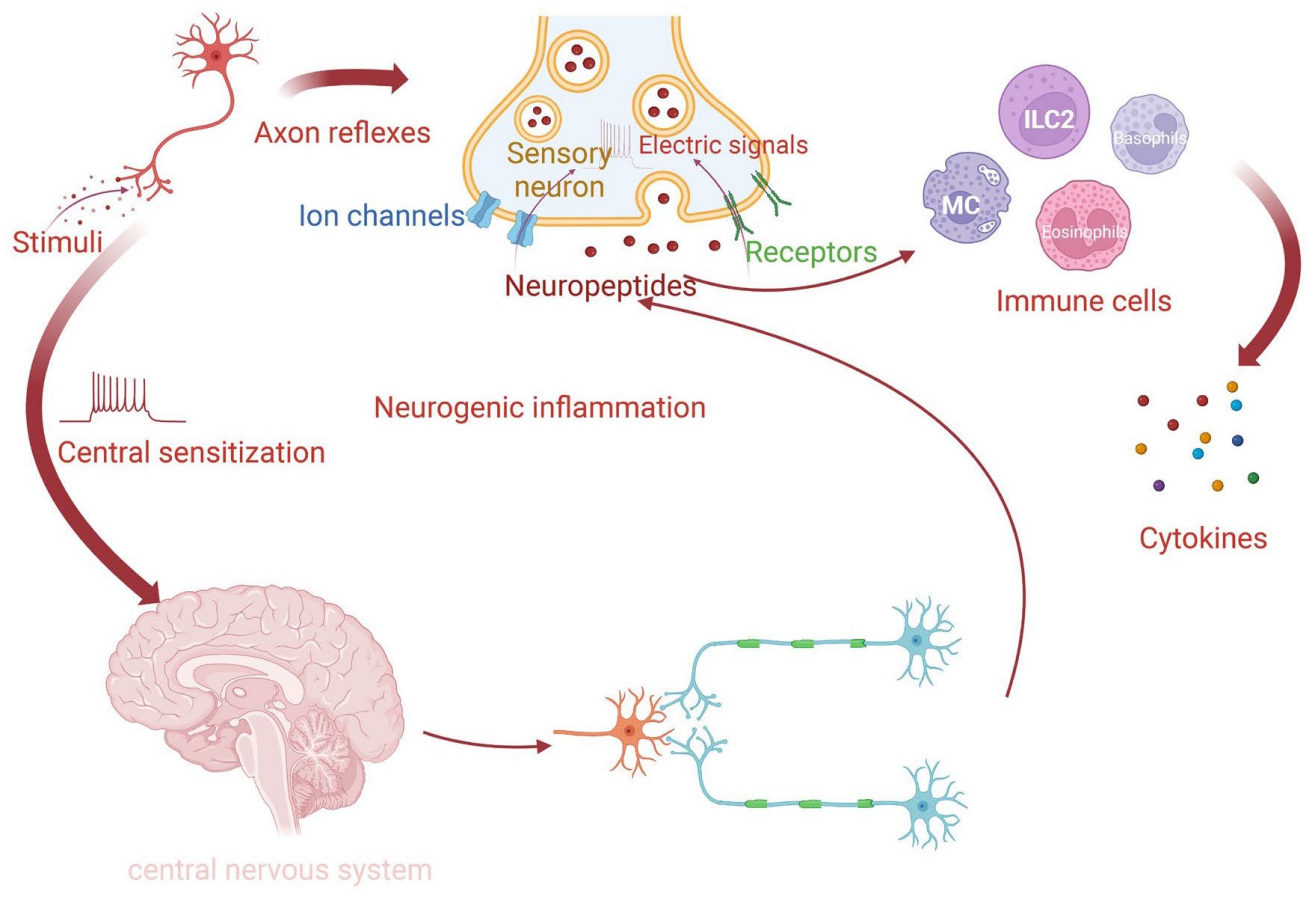
The process of neurogenic inflammation in AR. For sensitive individuals, when nasal nerve endings detect routine stimuli, they can transform environmental signals into electrical signals via various receptors and ion channels. There are two pathways to form neurogenic inflammation. One is called local “axon reflexes,” which releases neuropeptides through local afferent-efferent synapses independently. Another way is “central sensitization,” in which signals integrate in the central nervous system (CNS). The release of neuropeptides is facilitated by both of these mechanisms and then promotes different immune cells to produce cytokines.

Previous studies have demonstrated that various proinflammatory neuropeptides, including CGRP, SP, neuromedin U (NMU), nerve growth factor (NGF), and vasoactive intestinal peptide (VIP), are released by different nerve endings, especially sensory nerves in the nasal mucosa, in response to surrounding environmental stimuli. The exposure to allergens stimulates human C-fibers, resulting in a local peripheral reflex in sensitive individuals. The signal in this reflex is not integrated into the central nervous system (CNS) but is transduced via local axons (hence called “axon reflexes”). The action potentials generated in the peripheral sensory nerve endings are concentrated and transmitted until they reach the bifurcation, where they reverse back to other peripheral terminals of the same nerve. The afferent-efferent synapses of nasal mucosa promote neuropeptide release and immune cell recruitment, which can augment allergic inflammation. “Central sensitization” is mediated by integrating the reflex signal with the CNS (21, 27–31). Both these pathways can induce “neurogenic inflammation” and promote NHR by recruiting and activating immune cells (29, 32) (Figure 1). Nerves and type 2 cytokines, including interleukin (IL)-4, IL-5, and IL-13 can contribute to the neurological symptoms of AR, such as itching and sneezing. Type 2 cytokines induce neurological symptoms by activating sensory nerves. In addition, it has recently been discovered that thymic stromal lymphopoietin (TSLP) in mice binds to its receptor, TSLPR, activating TRPA1 (33). IL-31 produced by Th2 cells activates TRPV1+ TRPA1+ sensory nerves in animal models, inducing an MC-independent itching response (34–37). Additionally, IL-33 promotes itching through the receptor ST2 in peripheral TRPV1-expressing sensory neurons in mice. This is a histamine-independent way to activate Ca2+ influx into neurons (38, 39). Histamine upregulates mucin production in AR through the activation of cholinergic nerve terminals and cysteinyl leukotrienes (22).

### 2.3 Role of TRP in AR

Members of the ion channels known as the transient receptor potential (TRP) family, which link the nervous system with the immune system, are frequently expressed on afferent C-fibers. TRPV1 and TRPA1 mediate neurogenic and immunogenic inflammation associated with common respiratory illnesses, including AR. Additionally, TRPs are expressed on the surface of immune cells, such as MCs. In combination with activated MCs, activated TRPA1 and TRPV1 in mice mediate neuroimmune communication, inducing airway allergy symptoms through interaction with sensory nerves (35). The opening of the TRP channel promotes the influx of cations, such as sodium and calcium, generating action potentials and promoting the release of neuropeptides, such as SP and CGRP from the nasal mucosa to induce neurogenic inflammation. Additionally, TRP channels increase proinflammatory gene expression, vasodilation, capillary permeability, and plasma extravasation and regulate MC functions (35–37, 40, 41).

TRPA1, a nociceptive cationic (mostly Ca2+) thermo-responsive channel, is expressed in the trigeminal sensory neurons, which innervate the respiratory and nasal epithelia. The *TRPA1* mRNA level is upregulated in the nasal mucosa of patients with AR. TRPA1 is involved in the perception of cold temperatures (<17°C) (40, 42, 43). Low temperatures are reported to activate sensory nerves and induce neurogenic inflammation. This can explain the reason for cold air-stimulated sneezing in patients with AR. In the MC line RBL2H3, TRPA1 interacts with the granulogenesis-related protein secretogranin III in vesicular structures. Prostaglandin D2 and its metabolites can gate TRPA1 and strongly activate C-fibers (44). Recent studies have demonstrated that the inhibition of TRPA1 with the pharmacological antagonist HC-030031 alleviates nasal hyperresponsiveness and allergic inflammation of the upper airways in the ovalbumin-induced AR mouse model. The underlying mechanism may involve the TRPA1-SP signaling pathway (43). Thus, the inhibition of TRPA1 is a potential therapeutic strategy for patients with AR.

Receptors of TRPV1, which is a receptor of capsaicin that is directly activated by thermal, chemical, and mechanical stimuli, are associated with histamine-dependent arachidonic acid metabolite-induced itching (45). The density of TRPV1 on the surface of sensory fibers was upregulated in patients with hyperresponsiveness. Additionally, TRPV1 is widely distributed in the submucosal glands, vascular endothelial cells, and nasal mucosa. Inflammatory mediators can activate TRPV1 in the airway mucosa to induce inflammation (41, 46). The proximity of MCs to the sensory nerve fibers expressing TRPV1 facilitates nerve-MCs interactions (47). In AR mice, TRPV1 can express on the surface of CD4+ T cells and TRPV1 knockout downregulates the levels of infiltrating eosinophils, Th2/Th17 cells in the nasal mucosa, and specific IgE in the serum. TRPV1 is associated with AR clinical symptoms, especially itching and sneezing (48). Acupuncture treatment alleviates the symptoms of AR mice by modulating TRPV1 expression (49). Treatment with the TRPV1 agonist capsaicin alleviates cold dry air-induced symptoms and hyperreactivity in humans (22, 50, 51). Further clinical trials are needed to verify the therapeutic effectiveness of capsaicin in AR mediated through the TRPV1-SP nociceptive signaling pathway. Additionally, Chinese medicines exert therapeutic effects on AR through TRPV1 (41), although the underlying mechanisms have not been elucidated.

## 3 Role of neuropeptides in neuroimmune communication in AR

Several immune cells express receptors for the neuropeptides released from nerves, including acetylcholine, NGF, catecholamines, SP, NMU, VIP, and CGRP, which exert regulatory effects on various immune cells (34, 52). Additionally, some neuropeptides are secreted from the immune cells in AR.

### 3.1 Substance P

SP, a member of the tachykinin family of neuropeptides, is upregulated in the CNS, peripheral nervous system, and the immune system, has been found to have dramatically elevated levels in AR, and primarily operates through the neurokinin 1 receptor (NK1R) (11, 53). The expression of NK1R is upregulated in patients with AR (54). NK2R and NK3R are also SP receptors, however, their affinity is much lower than that of NK1R (53, 55, 56). Recent research has shown that SP, in addition to the classical receptor NK-1R, stimulates the MRGPRX2 receptor (and its murine counterpart, MRGPRB2) to produce allergies (57–59). SP exerts regulatory effects on immune cells, inducing vasodilation, increasing vascular permeability, and stimulating the submucosal glands in patients with AR (60). The SP levels are upregulated in the nasal lavages of individuals with AR. Additionally, the number of SP-positive nerve fibers was upregulated in individuals with NHR, suggesting the regulation of SP during neurogenic inflammation (61). The expression of the capsaicin receptor TRPV1 on the surface of all SP-positive sensory neurons indicated the functional role of TRPV1 in SP synthesis and release (61). Acupuncture treatment markedly downregulated allergen-specific IgE and SP and alleviated symptoms, especially those that are mediated by TRPV1, such as eye itching, nasal itching, and sneezing (49, 62).

The therapeutic strategies for AR patients targeting the nerves include selective vidian neurectomy with amputation of the posterior nasal nerve and pharyngeal branch of the vidian nerve, vidian neurectomy, radiofrequency neurolysis of the posterior nasal nerve area, and acupuncture at the sphenopalatine ganglion (63–65). These therapies may target various nerves. However, all of these nerves are branches of the trigeminal nerve. The trigeminal ganglion’s sensory neuronal cell bodies and afferent nerve terminals are where SP is concentrated. The production of SP in trigeminal ganglion neurons (TGNs) after allergen stimulation was 500-fold higher than that in nasal epithelial cells, indicating the capacity of TGNs to synthesize SP (66).

The balance between Th17 and Treg cells may be crucial for the pathogenesis of AR (10). The differentiation of Th17 is closely associated with both IL-6 and tumor growth factor (TGF)-β. Treg cells are correlated with the IL-10 levels (67). SP regulates the expression of IL-6 and TNF-α in MCs through the nuclear factor (NF)-κB pathway or the MyD88 pathway. The levels of SP are positively correlated with those of IL-6 and TNF-α but negatively correlated with those of IL-10 (11). Future studies must elucidate the underlying mechanism. SP is reported to promote dendritic cell (DC) migration through MRGPRA1. TRPV1+ sensory neurons and SP-driven DC migration are druggable pathways in type-2 immune response (68). NK1R-signaling is critical for efficient Ca2+ flux in T cell receptor-activated T cells. The deficiency or the pharmacological inhibition of NK1R on the surface of T cells downregulated Ca2+ flux (69). Additionally, SP can directly promote Treg dysfunction and induce the release of IL-17 by human memory-phenotype T cells through NK1R. These effects are successfully eliminated when SP-NK1R signaling is blocked in mice (70). These findings indicate that SP-mediated Th17/Treg differentiation induces AR.

**Figure 2:**
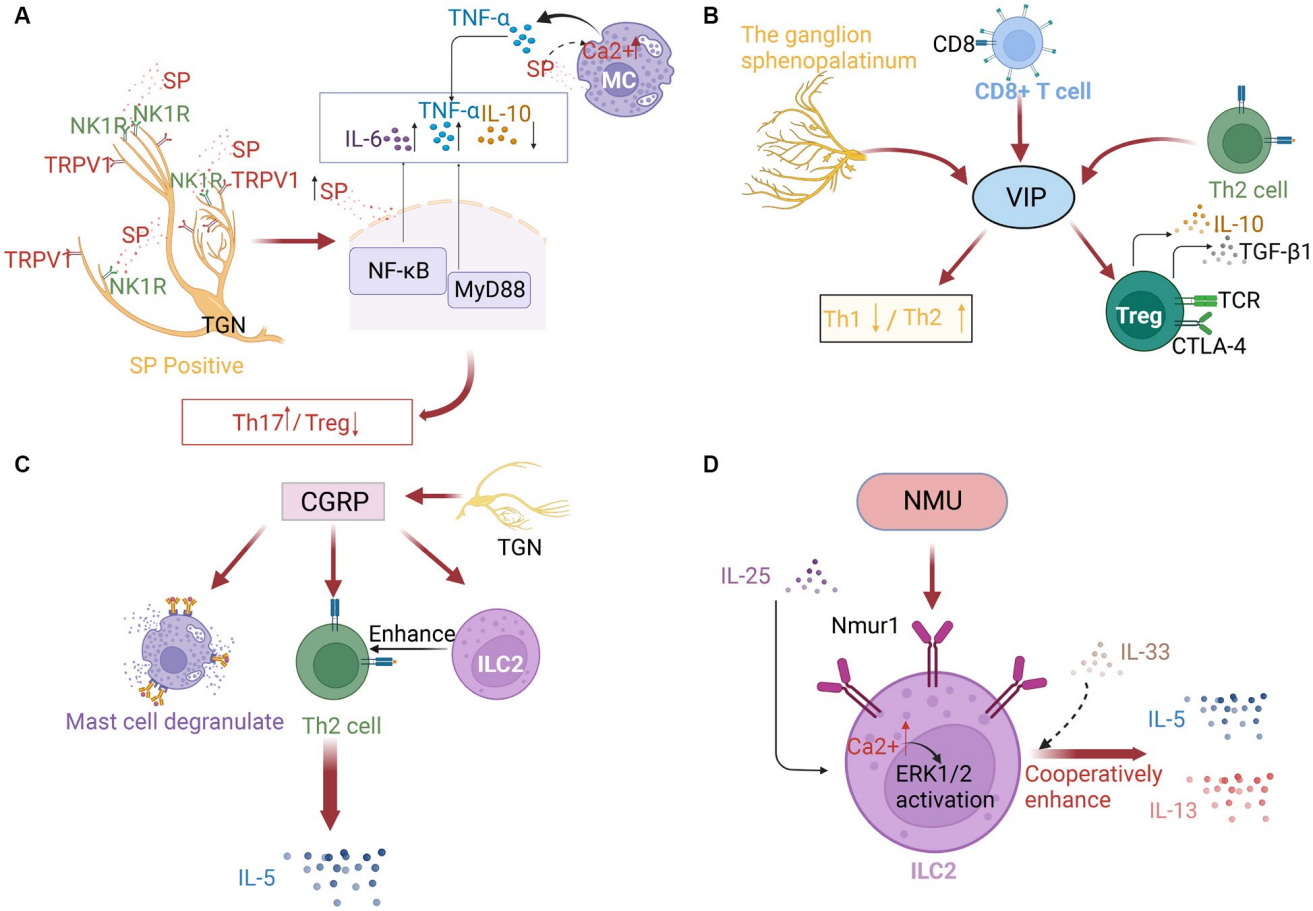
**(A)** Substance P (SP) in neuroimmune communication in AR. The trigeminal ganglion’s sensory neuronal cell bodies and afferent nerve terminals are where SP is concentrated, which are frequently TRPV1-positive. After binding to the neurokinin 1 receptor (NK1R), SP may control neurogenic inflammation via the NF-κB pathway or the MyD88 pathway. As SP concentrations increased, IL-6 and TNF-α were elevated while IL-10 was downregulated. By increasing the intracellular [Ca2+] of mast cells, SP can also stimulate TNF-α production. **(B)** Vasoactive intestinal peptide (VIP) in neuroimmune communication of AR. The ganglion sphenopalatinum is the origin of VIP. It can also be secreted by immune cells, especially by type 2 CD8+ T cells and Th2 cells. It can regulate Th1/Th2 balance, and promote Treg cell release of IL10 and TGF-β1. **(C)** Calcitonin gene-related peptide (CGRP) in neuroimmune communication of allergic rhinitis. Massive amounts of CGRPs are secreted by TGNs. They can trigger mast cell degranulation, ILC2 proliferation, and the following Th2 response, which induces the production of IL-5. **(D)** Neuromedin U (NMU) in neuroimmune communication of allergic rhinitis. ILC2s preferentially express neuromedin U receptor 1 (Nmur1), especially after IL-25 stimulation, and with the enhanced Ca2+ influx and the subsequent ERK1/2 activation, IL-5 and IL-13 expression, with IL-33 or independently of IL-33, is also increased.

In addition to nerve cells, MCs and eosinophils can synthesize and release SP. SP promotes TNF-α secretion from MCs. Additionally, the SP-induced Ca2+ influx affects the degranulation mechanism of MCs. Furthermore, SP induces histamine release from MCs (71, 72) (Figure 2A). McCary reported that SP can downregulate the expression of the high-affinity IgE receptor (FcεRI) mRNA and protein on the surface of human MCs, which in turn limits MC activation (73). This may contradict the ability of SP to activate MCs or indicate the presence of an SP-mediated non-IgE-dependent MC activation mechanism. Recent studies on non-IgE-mediated allergy reactions have revealed the role of SP in MC activation (59, 74–76). SP elicits inflammatory responses through MRGPRX2, challenging our previous understanding of the SP/NK1R axis (74). The intricate mechanism must be further investigated. Additionally, Sch 50971, a potent and highly selective H3 receptor agonist, alleviates nasal allergy symptoms in mice by inhibiting the production of SP through ATP-sensitive K+ channels (77).

In addition to its effect on MCs, recent studies have demonstrated that a burst of neuronal and epithelial SP serves as an initial defensive response in the nose (66). A recent study on patients with AR demonstrated that the SP-Toll-like receptor axis is impaired with delayed and prolonged upregulation of TLR4, which contributes to the more severe and persistent responses to infection in AR (78). This may aggravate nasal symptoms of AR, especially when encountered with infections.

### 3.2 Vasoactive intestinal peptide

VIP is mainly produced by parasympathetic nerves, and the sphenopalatine ganglion is the origin of nerve fibers in the upper respiratory tract that contain VIP. Additionally, VIP is additionally secreted by a variety of immune cells, including MCs, eosinophils, lymphocytes, and primarily type 2 CD8+ T cells and Th2 cells, especially under inflammatory conditions or after antigenic stimulation (79–81). VIP can maintain the Th1/Th2 balance by downregulating inflammatory Th1 type immune responses and upregulating Th2 type immune responses. Additionally, VIP-induced CD4+ CD25+ cells exhibit an active Treg cell phenotype and release high quantities of IL10 and TGF-β1 (79, 80) (Figure 2B). Compared with those in the nasal mucosa of healthy subjects, the mRNA and protein levels of VIP receptors were upregulated in the nasal mucosa of patients with AR. Additionally, the nasal tissue and secretion levels of VIP in patients with AR were upregulated when compared with those in healthy individuals. The number of VIP-positive fibers was significantly upregulated in the nasal tissues of patients with AR (81). Acupuncture treatment in AR patients significantly downregulated the salivary VIP levels (49). VIP can stimulate the secretion of human serous cells, expand the nasal blood tube, and regulate the clearance of nasal mucosa cilia (82, 83). The effects of VIP on the AR are mediated through their specialized receptors (CRTH2, PAC1, VPAC-1, and VPAC-2), which are expressed on various immune cells, including MCs, eosinophils, and lymphocytes (79, 84). These receptors mediate the regulatory effects of VIP on immunological response through various protein kinases, including the phospholipase C/PKC and mitogen-activated protein kinase (MAPK) pathways, as well as the adenylate cyclase/PKA pathway. On inflammatory cells like basophils, eosinophils, and Th2 lymphocytes, the CRTH2 receptor has been identified. Blocking this receptor suppresses allergic airway inflammation (79). AR may be significantly influenced by the relationship between mast cells and the VPAC-2 receptor. The proximity of neurons expressing VIP to the immune system components and the expression of VIP receptors on the surface of immune cells indicated the VIP-immune system interaction in AR.

### 3.3 Calcitonin gene-related peptide

The neuropeptide CGRP is massively secreted by TGNs. The expression of CGRP has been detected in 50% of human TGNs. CGRP, which is released from both peripheral nerve and central nerve terminals, exerts various biological effects, especially vasodilation (71). In patients with AR, allergen challenge promotes CGRP-mediated nasal congestion and upregulates mucosal edema caused by histamine and lipids. The level of CGRP is significantly upregulated in nasal secretions. The alleviation of nasal symptoms in AR patients is associated with the downregulation of CGRP in the nasal mucosa (85, 86). MCs are frequently detected near CGRP-expressing nerve fibers. CGRP regulates the expression of several genes involved in nerve fiber activation. Additionally, CGRP promotes the recruitment of MCs in mice (87). Animal studies have demonstrated that CGRP induces MCs degranulation and facilitates Th2 differentiation (34, 88). The direct effect of CGRP on innate lymphoid cells (ILCs) stimulates a Th2 response (71), which may promote the development of AR.

CGRP stimulates the expression of nuclear factor of activated T cells c2(NFATC2) through the cAMP/PKA pathway by binding to the activity-modifying protein 1 (RAMP1) and calcitonin receptor-like receptor (CLR), promoting the production of IL-17 in Th17 cells. This mechanism is suppressed in RAMP1-deficient mice (89). Recent studies have demonstrated that the CGRP-ILC2 axis contributes to allergic asthma development, and may be crucial in the initiation of AR type 2 inflammation. CGRP can act directly to stimulate the maturation of ILC2s and promote the generation of cytokines, such as IL-5. ILC2s that produce IL-5 have critical roles in the development of allergy symptoms. Activated ILC2s promote the recruitment of eosinophils and initiate a cascade of Th2 responses. These ILC2s can be suppressed upon treatment with CGRP antagonists (Figure 2C) (90, 91). Targeting ILC2 to inhibit the CGRP signaling is a novel therapeutic strategy for allergy disorders.

### 3.4 Neuromedin U

Cholinergic neuron-derived NMU, a 33 amino-acid peptide that is widely conserved, functions as a rapid and effective type 2 cytokine regulator (92). NMU and its receptor NMUR1 are expressed in T cells, DCs, eosinophils, and MCs. MCs may regulate the NMU/NMUR axis in humans (93). NMU promotes Ca2+ mobilization and degranulation in MCs, inducing vasodilation and plasma extravasation (94). Previous studies have demonstrated that NMU stimulates ILC2s and consequently induces the production of type 2 cytokines in enteritis and asthma mouse models (95, 96). After allergen exposure, NMU functions as a neuronal amplifier to elicit a strong, rapid, and selective activation of ILC2s (96). ILC2s preferentially express NMUR1, especially after IL-25 stimulation, while mucosal neurons express NMU. The co-administration of NMU and IL-25 markedly exacerbated allergic inflammation *in vivo* (95, 97). NMU cooperatively enhanced IL-5 and IL-13 expression through IL-33-dependent or IL-33-independent mechanisms (96, 98). A recent study in AR patients demonstrated that NMU activates ILC2s, resulting in the upregulation of the inflammatory factors IL-5 and IL-13 rather than IL-33 in patients with AR, triggering a type 2 inflammatory response, which was inhibited upon ERK pathway blockade (99).

These findings indicate the presence of a unique neuroimmune pathway that can exacerbate allergic inflammation of the mucosa and induce nasal hyperresponsiveness (97). The induction of MC degranulation is critical for AR pathogenesis (100). ILC2s, which express NMUR1, are associated with neurons that express NMU to generate neuroimmune cell units (92). The mechanism may involve NMUR1-mediated NMU stimulation of ILC2s, resulting in enhanced Ca2+ influx, effective ERK1/2 activation, and cytokine generation (95) (Figure 2D).

### 3.5 Nerve growth factor

NGF was the first member of the neurotrophin family protein to be discovered. The main function of NGF is to promote the growth, differentiation, and survival of peripheral and central nerves (101). NGF, which is expressed in the nasal epithelium, glandular epithelium, and peripheral nerves in the nasal mucosa, plays a critical role in the bidirectional signaling mechanisms between the neurosensory network structures and immune cells that can lead to hyperresponsiveness (1, 102, 103). In patients with AR, the NGF protein level is upregulated in the nasal submucosa, submucosal glands, nasal lavage fluids, and serum. NGF is localized to the MCs and eosinophils of the nasal mucosa (4, 6, 103, 104). The levels of NGF-positive nerve bundles, tropomyosin receptor kinase A (TrkA), and p75 receptors of NGF are significantly upregulated in patients with AR (1), indicating the role of NGF in the allergic inflammatory process of AR.

In addition to neurons, immune cells, including MCs and eosinophils, can release NGF (29, 104). TrkA and p75 receptors are expressed in the nerves, the nasal epithelium, and submucosal glands (1). MCs that express and secrete NGF are close to the tachykinin-containing nerve terminals in the peripheral tissue. NGF, which upregulates the expression of neuropeptides, such as SP, regulates the transit of SP in sensory nerves to promote the antidromic release of SP upon nerve activation (6, 104). In the human nasal mucosa, TrkA and p75 receptors were identified along the sensory nerves (4). These characteristics favor the formation of neuroimmune regulatory units, revealing neuroimmune interaction in AR-associated NHR.

**Figure 3:**
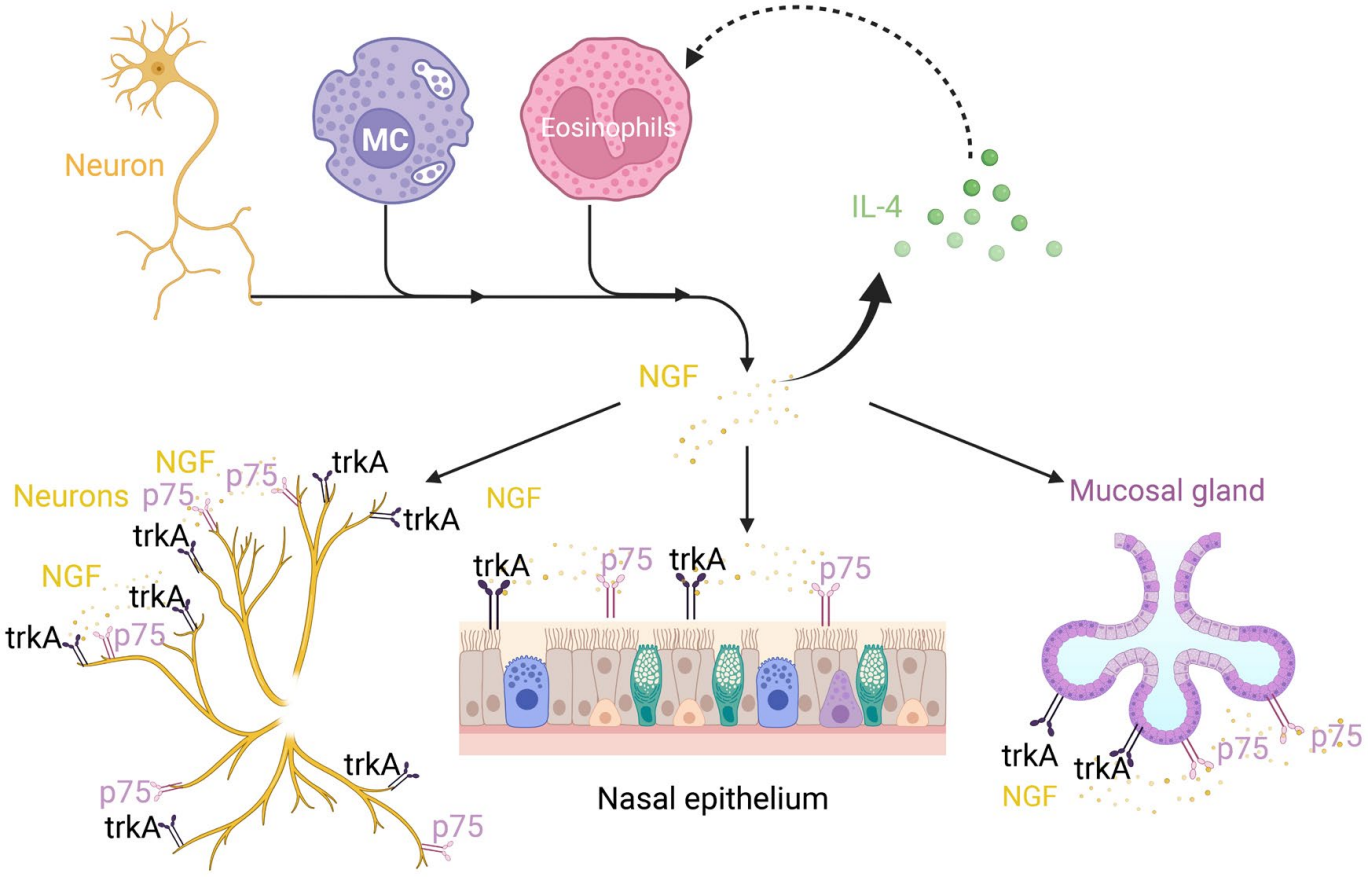
Nerve growth factor (NGF) in neuroimmune communication in AR. In addition to neurons, mast cells and eosinophils can also release NGF. The TrkA and p75 receptors can not only express on neurons but also on the nasal epithelium and submucosal glands. This can boost IL-4 secretion, which in turn promotes NGF secretion by eosinophils.

A recent study reported that the prolonged stimulation of isolated sensory neurons by NGF promotes a switch from PKA activation to Epacs activation in the signaling pathway, resulting in prostaglandin E2-induced hypersensitivity (105). Exposure to cold temperatures upregulates the number of eosinophils and the production of NGF in mice, which promotes sympathetic axonal outgrowth. In both humans and mice, sympathetic nerves are located close to IL-33-expressing stromal cells and eosinophils. The signal from sympathetic nerves triggers calcium flux in stromal cells and the subsequent release of IL-33, which upregulates IL-5 in ILC2s (106). Additionally, NGF directly affects eosinophils by promoting IL-4 secretion (1) (Figure 3). These immediate and delayed effects constitute the mechanism through which NGF mediates AR inflammation and hyperresponsiveness (1).

## 4 Formation of neuroimmune cells units in AR

Immune and neural cells are frequently colocalized at certain anatomical locations. Eosinophils, lymphocytes, and MCs accumulate in neuroimmune cell units around the airway nerves, where they coordinate their responses for bidirectional neuroimmune interactions (107–109). The number of neuroimmune cell units is upregulated in patients with AR.

### 4.1 Nerve-MCs unit

#### 4.1.1 Role of MCs in AR

The high-affinity IgE receptor FceR1 is expressed on the surface of MCs (110). Various inflammatory mediators, including eicosanoids, proteases, biogenic amines, chemokines, and cytokines, are released upon allergen-mediated activation of MCs through the cross-linking of cell surface-bound IgE to its high-affinity receptor (FcεRI) in sensitized individuals, exacerbating allergic inflammation (111, 112). Additionally, the binding of IgE to FceR1 stabilizes the expression of the receptor on cell surfaces, upregulating the expression of FceR1 (113). Histamine, leukotrienes, and tryptase are MC-derived mediators that interact with specific receptors on sensory nerve endings, promoting the production of neuropeptides (74). MCs can also respond to stimulation with neuropeptides in an FcεRI-independent manner. These findings indicate the function of MCs in neuroimmune interactions (79).

Tryptases and chymases, which are proteases expressed in MCs, are regulated by IL-10 and IL-4 (111). MCs can be classified based on the contents of their secretory granules as follows: MCTC, granules contain both tryptase and chymase; MCT, granules contain tryptase but little or no chymase; MCC, an infrequent phenotype and the granules contain little chymase or no tryptase (114–116). Consequently, only MCT and MCTC are referenced in some studies. In patients with AR, the tryptase levels are upregulated in the nasal lavage and the phenotype of MCs resembles that of MCT. The serum tryptase levels were associated with the levels of neuropeptides, indicating that neuropeptides are involved in the regulation of MCs (81, 116).

#### 4.1.2 Nerve-MCs colocalization and communication with neuropeptides

MCs and neuropeptide-containing nerves are anatomically connected and are prevalent in patients with AR. The MC-mediated immune reaction is downregulated in the absence of sensory nerves (34, 117, 118). The anatomical distance between MCs and nerves enables bidirectional communication. A small number of MCs form synaptic-like contacts with nerves that are dependent on integrins, especially cell adhesion molecule 1 (CADM1; also known as SynCAM1). CADM1, which is expressed in MCs and sensory neurons, mediates interactions between MCs and sensory neurons. IL-6 production and degranulation are activated in MCs through CADM1-dependent interactions with sensory neurons. CADM1 knockdown in MCs markedly impaired the physical interaction and the functional relationship with sensory neurons in mice (119). Intragranular particles of MCs can be transported into neurons through direct fusion with the plasma membrane of the neuron or through the neuronal capture of insoluble granule remnants (120).

**Figure 4:**
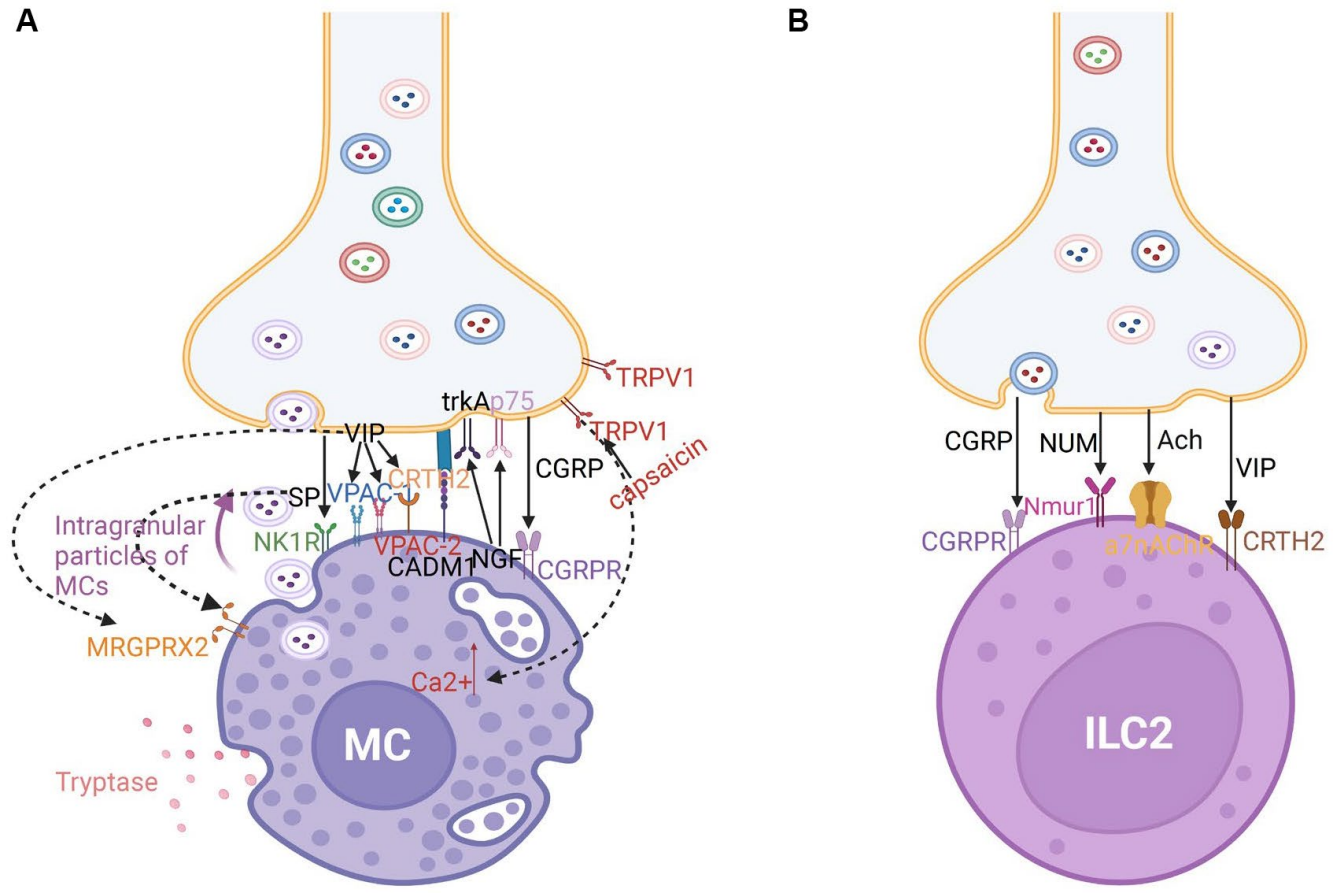
**(A)** Nerve-MC unit in neuroimmune communication in allergic rhinitis. Tryptase-secreting MCs play a significant role in allergic rhinitis. The development of nerve-MC regulating units is mediated by CADM1. Intragranular particles of MCs can be transported into neurons. There are a large number of receptors on MCs such as calcitonin-receptor-like receptor (CGRPR) for CGRP; NK1 and MRGPRX2 receptors for SP; VPAC-1, VPAC-1, MRGPRX2, and CRTH2 receptors for VIP; and TrkA and p75 receptors for NGF. Capsaicin stimulates TRPV1, which encourages mast cell Ca2+ increase and the ensuing immunological response. **(B)** Nerve-ILC2 unit in neuroimmune communication in allergic rhinitis. ILC2s express CGRPR for CGRP, Nmur1 for NUM, a7nAChR for Ach, and CRTH2 receptors for VIP.

Human MCs express a CGRP receptor (CGRPR), SP receptor (NK1R), VIP receptors (VPAC-1 and 2), and Mas-related gene receptor X2 (MRGPRX2), a cation-sensing receptor for various ligands such as SP, VIP and somatostatin (SST) (121). MRGPRX2 mediates non-IgE-dependent activation in patients with AR, inducing hypersensitivity, neurogenic inflammation, and itching (122). Additionally, MCs promote the release of neuropeptides, such as SP and CGRP from nerves (117). Trypsin and MC tryptase activate the protease-activated receptor 2 (PAR2), which is expressed on the surface of nerve fibers, including trigeminal neurons in the human nasal mucosa, eliciting a generalized inflammatory response through unidentified pathways. Additionally, trypsin and tryptase promote the release of SP and related peptides from sensory neurons, mediating neurogenic inflammation (123, 124). The CRTH2 receptor is a G protein-coupled receptor for the Prostaglandin D2 receptor. It was discovered that VIP activates the synthesis of the CRTH2 receptor, which is expressed by 34% of mast cells in human nasal polyps. Recruitment of inflammatory cells is induced by this interaction. Furthermore, blocking this receptor decreases allergy manifestation (79) (Figure 4A). In addition, it has been demonstrated that nerve–MC interactions allow corticotropin releasing hormone (CRF) and associated peptides to regulate ion and water secretion. C nerve fibers express H1 and H4 receptors for histamine, and those activated nerves release neuropeptides which in turn induce histamine and prostaglandin D2 release from MCs (125). These findings revealed that MCs serve as a functional homeostatic regulatory unit to facilitate neuroimmune interaction.

### 4.2 Nerve-ILC2s unit

Innate lymphoid cells (ILCs) without receptors to recognize antigens like T and B cells are novel immune cells with a lymphoid appearance and are abundantly distributed in the mucosal tissues. Additionally, ILCs have been divided into three different subpopulations. ILC2s share phenotypic characteristics with Th2 cells (126). Various cytokines, especially IL-25 and IL-33, can induce ILC2s to release type 2 cytokines, such as IL-13 and IL-5 to trigger allergic tissue inflammation at the mucosal surfaces in patients with AR (97, 127). Recent studies on the communication between nerves and ILC2s revealed that type 2 inflammation in allergy disorders is significantly influenced by neuroimmune interactions (34). Neurons, especially cholinergic neurons in mice, form close intercellular contacts with ILC2s, generating a novel neuron-ILC2 unit (95, 96, 98).

Cholinergic neurons release the neuropeptide NMU, which promotes type 2 cytokine responses through ILC2 activation. Compared with that in other ILC subtypes, the expression of NMUR1 is upregulated in ILC2s. Additionally, CGRP modulates the activation of ILC2s, which regulate the intensity of type 2 inflammation in mice (95, 96, 128). The a7-nicotinic acetylcholine receptor (a7nAChR) is expressed by ILC2s in mice (129) (Figure 4B). The agonists act on a7nAchR and may suppress phosphorylation of upstream kinase IKKa/b and NF-kB expression to reduce the release of cytokines like IL-5 and IL-13 and suppress airway hyperreactivity (AHR). Acetylcholine may inhibit ILC2-mediated inflammation through a7nAchR (127, 129).

Most neuropeptides bind to GPCRs, which comprise alpha, beta, and gamma subunits. G alpha proteins are categorized into Gas, Gai, and Gaq subtypes. Gas activates adenyl cyclase (AC), which upregulates intracellular cAMP levels and activates PKA, enhancing the cellular Ca2+ concentration. Gai inhibits AC activation and decreases intracellular cAMP levels. Intracellular Ca2+ induces ILC2s to trigger a cascade of Th2 responses. In contrast, cAMP inhibits ILC2 activation. Therefore, the type of Ga protein, which can exert different effects on intracellular Ca2+ and cAMP levels, can be used to elucidate the regulatory mechanisms of neuropeptides in ILC2s. Catecholamines suppress ILC2-mediated inflammation by upregulating cAMP levels through Gas protein (127).

### 4.3 Nerve-eosinophils unit

**Figure 5:**
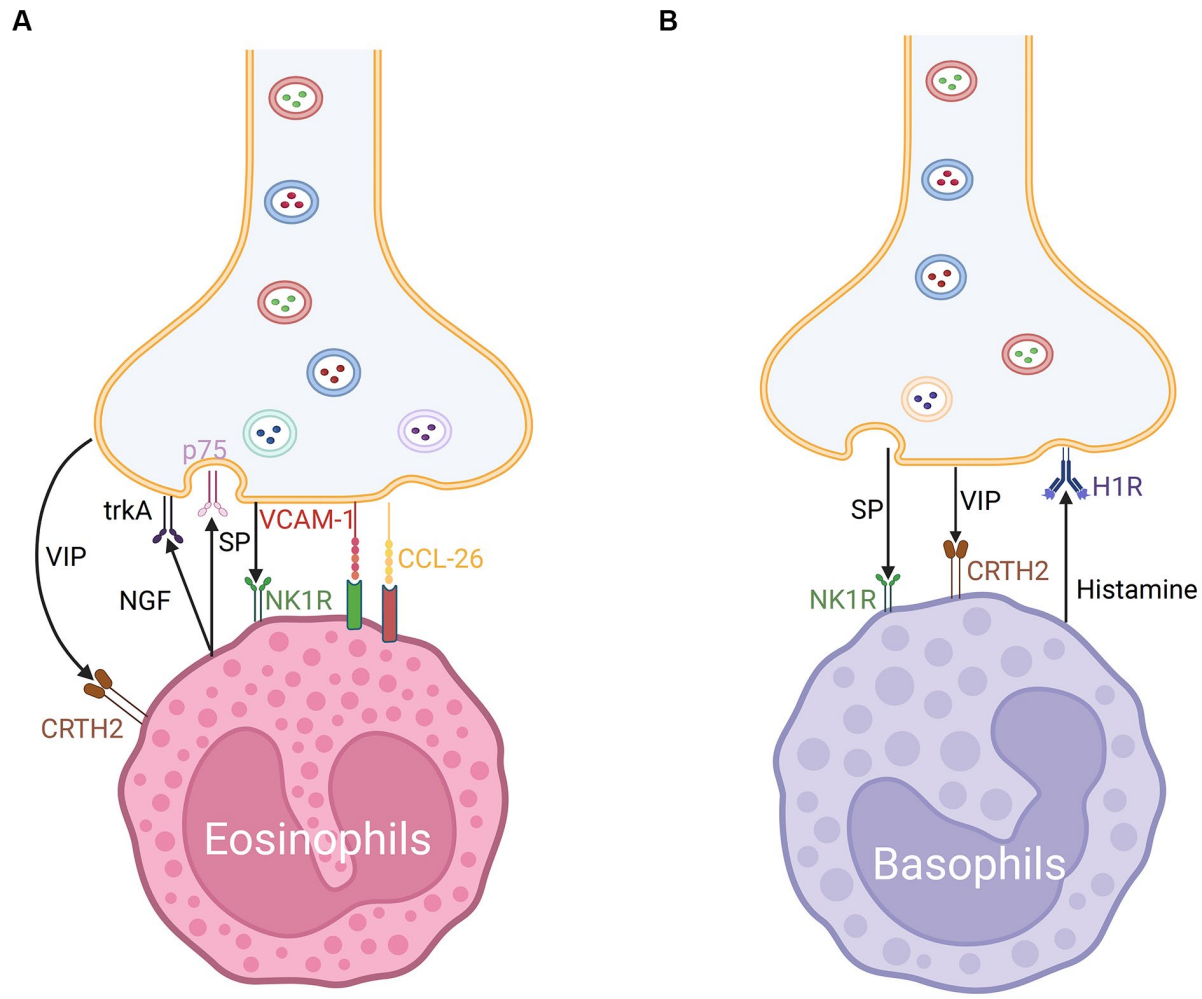
**(A)** Nerve-eosinophils unit in neuroimmune communication in allergic rhinitis. The co-localization of nerve fibers and eosinophils may be mediated by CCL-26 and VCAM-1. Eosinophils express CRTH2 receptors for VIP and NK1 receptors for SP. Eosinophils can secrete NGF which acts on trkA and p75 of nerves via synaptic-like contacts. **(B)** Nerve-basophils unit in neuroimmune communication in allergic rhinitis. Basophils produce histamine which acts on the H1R of nerve fibers and expresses NK1 receptors for SP and CRTH2 receptors for VIP.

Eosinophils are recruited to nasal nerves after an allergen challenge through the reduction of neuron activation threshold. The recruitment of eosinophils promotes nerve growth and modulates neuropeptide production, resulting in neuronal hyperreactivity in AR (109, 130). Inhibitory M2 muscarinic receptors (M2Rs) expressed on the surface of postganglionic nerves in experimental animals can be blocked by basic protein (MBP) expressed in activated eosinophils, promoting acetylcholine release (131). Eosinophils tend to colocalize with the choline acetyltransferase (ChAT) immunoreactive nerves. In patients with AR, eosinophils exhibit CRTH2 expression (109). Previous investigations in AR patients showed that VIP peptide and CRTH2 molecules co-localized and had a significant interaction (83). The co-localization may be mediated by eosinophil chemoattractant CCL-26 and VCAM-1 expressed by nasal nerve fibers in animal models (109). In combination with other mechanisms, including SP immunoreactive nerves, this leads to hyperresponsiveness of the nasal nerves, contributing to NHR development (109) (Figure 5A).

The spraying of glucocorticoids targeting eosinophilic inflammation is the main drug for treating AR and is currently considered relatively safe (132). Additionally, in antigen-induced individuals, neutralizing MBP with a specific antibody or with polyanionic substances such as heparin or poly-l-glutamic acid and reducing eosinophils with an anti-IL-5 antibody may prevent M2R dysfunction and nasal hyperresponsiveness. A CCR3 antagonist suppressed antigen-induced eosinophil recruitment along the nerves in animals, which may provide novel strategies for the treatment of AR (131).

### 4.4 Nerve-basophils unit

Basophils promote Th2 cytokine-mediated inflammation. The cross-linking of the high-affinity IgE receptor FcεRI and IgE on basophil surfaces after allergen challenge promotes the release of various mediators, which contribute to allergic inflammation in AR (133). Additionally, basophils were discovered in the nasal lavage fluid of AR individuals, which are considered to be the dominant source of histamine in anaphylactic late phase response (LPR). Therapeutics for AR may exert their effects on MCs and partially regulate basophil-derived histamine and leukotrienes (134).

Additionally, various immune inflammatory factors and neuropeptides modulate the function of basophils. Allergen-activated basophil-derived factors can directly activate and interact with sensory neurons. The basophil-neuronal circuit is involved in the pathogenesis of various neuroimmune processes associated with allergic reactions in AR, such as the formation of the basophil-leukotriene (LT) axis (135). The upregulation of MRGPRX2 was related to degranulation and CD63 expression in basophils in mice (122). CRTH2, a receptor of VIP, is also expressed on the surface of basophils and may mediate chemotaxis (79, 83) (Figure 5B). ILC2s from basophil-depleted mice express less neuromedin B (NMB) receptor, a member of the neuromedin family of neuropeptides that also includes NMU, NMK, NMC, NMS, NMN, and NML, demonstrating that basophils act as the transition switch necessary for ILC2s to react to NMB-mediated inhibition, which may work by modulating the expression of P2rx7 (128).

## 5 Potential roles of MRGPRX2 in neuroimmunomodulation in AR

MRGPRX2, originally discovered in neurons, is found not only in neurons but also in MCs and is expressed on comparable levels on peripheral blood basophils and eosinophils (122, 136, 137). The MRGPRX2-mediated activation of MCs is associated with neurogenic inflammation (138). Recent studies on MCs have focused on MRGPRX2, especially on its role in IgE-independent degranulation (116). Previous studies have reported that MRGPRX2 is mainly expressed in MCTC but not in MCT, while MCT is found on mucosal surfaces such as the lung (74, 116, 139). Although it was previously considered that mucosal MCs are of the MCT type and do not express MRGPRX2, it now appears that 20% of normal human lung mast cells express MRGPRX2 (74). Manorak et al. reported that the MRGPRX2 level was downregulated in non-asthmatic lung MCs but markedly upregulated in asthma lung MCs in humans (140). An et al. confirmed that the mean serum MRGPRX2 levels in patients with asthma were higher than those in patients without asthma (136). The serum MRGPRX2 levels in patients with chronic spontaneous urticaria were positively correlated with specific IgE against Dermatophagoides farinae, which is a major etiological factor for AR (141).

Ai J et al. discovered that endoscopic vidian neurectomy of AR improved QOL related to asthma in roughly half of the patients with AR (142). Imperatorin (IMP), which was recently demonstrated to be an inhibitor of MRGPRX2 and exert therapeutic effects on allergic asthma, exerts anti-inflammatory effects in the AR model (143). AR and asthma share pathogenesis and histological characteristics and are considered different manifestations of the same underlying atopic state. MRGPRX2 activation is hypothesized to significantly increase the inflammatory response in AR.

MRGPRX2 is suggested to contribute to the pathogenesis of AR by promoting IgE-independent activation of human MCs through neuropeptides (SP, NGF, CGRP, and VIP), cysteine proteases, antimicrobial peptides, and eosinophilic cationic proteins (137, 144). Treatment with MRGPRX2 ligands upregulates the calcium influx in human MCs (115, 145). SP causes itchiness by activating MRGPRX2 in addition to NK1R (59). In addition to SP, HK-1 stimulates human MCs via MRGPRX2 rather than NK1R, especially during airway inflammation (140). Selective MRGPRX2 antagonists are potential therapeutics for AR although further studies are needed to clarify their underlying mechanisms and enable their clinical application.

## 6 Psychological condition-mediated neuroimmune regulation in AR

Previous studies have reported a correlation between AR and psychological disorders (146). The neurons in the prefrontal cortex and olfactory bulbs of AR rats produce Th2 cytokines (147). The mechanism associated with IgE production includes cortisol-induced alterations in serotonin secretion mediated by the activated hypothalamic–pituitary–adrenal axis (HPA) due to hypersensitive response (146).

Several mediators released in AR can alter neuronal activity (especially in areas of the brain that process emotion and affective behaviors), activate the HPA axis, and induce stress response (148, 149). IL-1β can be generated by almost all nucleated cells, including B lymphocytes, natural killer cells, MCs, and epithelial cells (150, 151). Additionally, IL-1β directly acts on MCs and promotes the secretion of cytokines (152). Allergen exposure promotes IL-1β secretion in human nasal epithelial cells of patients with AR (153). IL-1β can also stimulate the HPA axis, promoting the production of cortisol, which triggers the release of serotonin and contributes to mood changes (146). The serum NGF levels are upregulated in caregivers who experience prolonged stress (154). Several studies have reported the correlation between allergic symptoms in children and the psychological state of caregivers (155). Warm and responsive caregiving can reduce the occurrence of allergic diseases in children (156).

## 7 Conclusion

Neuroimmune communication is reported to be involved in the pathogenesis of AR, which is now classified as a neuroimmune illness. Pharmacological agents that target neuroimmune communication can be potentially effective. Clinical evidence suggests that acupuncture can alleviate the symptoms of AR by downregulating SP, a proinflammatory neuropeptide (49). Vidian neurectomy (VN) or posterior nasal neurectomy (PNN) via endoscopy have been introduced to treat refractory AR and can significantly alleviate the symptoms of sneezing and rhinorrhea (157). Office-based cryotherapy of the PNN region can significantly decrease symptom scores (158). The effectiveness of nerve desensitization with repetitive application of capsaicin or resection of the vidian nerve indicated that the nasal mucosa of patients with AR exhibited nerve-immune mediated hyperresponsiveness (6). These treatments may affect neuroimmune interaction in AR. The development of nasal hyperresponsiveness is linked to immune cell modulation and reflexes. The formation of “axon reflexes” and “central sensitization” promotes the development of “neurogenic inflammation,” which stimulates the release of various neuropeptides from nerve endings and the recruitment and activation of immune cells, resulting in the development of symptoms. Several immune cells have receptors for neuropeptides. Immune cells also release neuropeptides, which exert regulatory effects. The SP levels in nasal lavages and the number of SP-positive nerve fibers in patients with AR were higher than those in healthy individuals. TRPV1 and TRPA1 are involved in SP-induced allergic immune responses. The concentrations of VIP and the number of VIP-positive fibers are upregulated in the nasal tissues of patients with AR. VIP maintains the Th1/Th2 balance through various receptors. TGN can secrete a large amount of CGRP, which induces the activation of both MCs and ILCs to elicit a Th2 response. NMU, NGF, and their receptors are crucial factors regulating neuroimmune communication in AR.

Immune cells are colocalized with nerves in specific anatomical locations, forming neuroimmune cell units. CADM1 mediates the formation of synaptic-like contacts in the nerve-MC unit, promoting bidirectional transport between MCs and neurons. TRPA1 and TRPV1 regulate the function of the nerve-MC unit. Nerve-ILC2 units are in the vicinity of cholinergic neurons and can regulate the severity of type 2 inflammation in AR. The nerve-basophils and nerve-eosinophils units are also involved in the pathogenesis of AR. Additionally, the inflammatory response in AR is exacerbated upon the activation of MRGPRX2, which is expressed in some immune cells and nerve cells. The silencing of MRGPRX2 downregulates MC degranulation. Analysis of the function of the HPA axis revealed that AR is correlated with psychological disorders.

The major therapeutic strategies for AR are drug therapy and immunotherapy. However, these therapies are associated with side effects, such as fatigue. Additionally, the adherence rate of immunotherapy is poor. The therapeutics for AR exhibit broad anti-inflammatory properties (typically glucocorticoids), neutralize secreted products (antihistamines), or inhibit IgE (omalizumab). However, these therapies are not effective in alleviating symptoms in some individuals. Thus, there is an urgent need to develop novel and efficient therapeutic interventions. The clinical application and potential mechanisms of surgical treatment and acupuncture targeting neuroimmune interaction must be examined in future studies. The TRPA1 antagonist HC-030031 and the TRPV1 agonist capsaicin were demonstrated to exert effective therapeutic effects on AR. However, the underlying therapeutic mechanisms of HC-030031 and capsaicin have not been elucidated. Additionally, although neuropeptides can be detected in nasal secretions, their clinical value has not been established. The neuropeptide levels in preformed nasal secretions can predict the treatment response of patients. Pharmacological products that counteract the effects of neuropeptides are promising therapeutic agents for AR. To suppress SP-induced itching, nociception, and inflammation in mouse models, various pharmacological NK1R antagonists have been developed. However, human trials with NK1R antagonists have yielded inconsistent results. Hence, novel SP receptor antagonists must be developed and their safety must be evaluated before clinical application. Neuroimmune communication may offer novel insights for developing diagnostic and therapeutic strategies. Hence, the ability of currently used therapeutics for AR to regulate neuroimmune communication must be investigated in the future.

## Author contributions

YZ: Writing – original draft, Writing – review & editing. RC: Writing – original draft. LK: Resources, Writing – original draft. YS: Resources, Writing – original draft. JD: Supervision, Writing – review & editing.
